# Hyperchloraemia in sepsis

**DOI:** 10.1186/s13613-018-0388-4

**Published:** 2018-03-27

**Authors:** Christos Filis, Ioannis Vasileiadis, Antonia Koutsoukou

**Affiliations:** 1 3rd Department of Internal Medicine, “Sotiria” Hospital, National and Kapodistrian University of Athens, 152 Mesogion Av., 115 27 Athens, Greece; 2 Intensive Care Unit, 1st Department of Respiratory Medicine, “Sotiria” Hospital, National and Kapodistrian University of Athens, 152 Mesogion Av., 115 27 Athens, Greece

**Keywords:** Hyperchloraemia, Chloride, Sepsis, Critically ill patients, Metabolic acidosis

## Abstract

Chloride represents—quantitatively—the most prevalent, negatively charged, strong plasma electrolyte. Control of chloride concentration is a probable major mechanism for regulating the body’s acid–base balance and for maintaining homeostasis of the entire internal environment. The difference between the concentrations of chloride and sodium constitutes the major contributor to the strong ion difference (SID); SID is the key pH regulator in the body, according to the physicochemical approach. Hyperchloraemia resulting from either underlying diseases or medical interventions is common in intensive care units. Recent studies have demonstrated the importance of hyperchloraemia in metabolic acidosis and in other pathophysiological disorders present in sepsis. The aim of this narrative review is to present the current knowledge about the effects of hyperchloraemia, in relation to the underlying pathophysiology, in septic patients.

## Background

Sepsis and septic shock affect over 26 million people worldwide and remain the leading causes of death in the US hospitals, despite progress in early recognition and treatment [1]. Acidosis is a frequently identified acid–base disorder in patients with sepsis and is linked to different pathophysiological routes (type II respiratory failure, renal failure, lactic acidosis, ketoacidosis). Chloride (Cl^−^) is the body’s major anion, representing two-thirds of all negative charges in plasma and is also responsible for one-third of plasma tonicity [2]. Its role and significance in acid–base balance, osmosis, muscular activity and immunomodulation has been overshadowed by other serum electrolytes, even though chloride abnormalities have been detected in 25% of patients in the critical care setting [3]. Aggressive fluid resuscitation, with chloride-rich crystalloids, during the treatment of sepsis-induced hypoperfusion, may lead to iatrogenic hyperchloraemic acidosis [4]. The present review focuses on the effects of hyperchloraemia in septic patients (metabolic acidosis, haemodynamics, inflammatory response, renal and gastrointestinal function, haemostatic disorders, mortality) and their underlying pathophysiology.

## Main text

### Hyperchloraemic metabolic acidosis

The major pathophysiological mechanisms leading to hyperchloraemic metabolic acidosis after fluid resuscitation with saline remain controversial. Van Slyke described the phenomenon of “dilution acidosis” almost 100 years ago [5], but the term was proposed after the Second World War [6]. He was the first to suggest that intravenous saline infusion leads to metabolic acidosis due to dilution of total body base. Further studies in non-intubated dogs showed that dilution acidosis was not related to the type of the solution used (0.9% saline, dextrose water 5%, mannitol 5%), since bicarbonate (HCO_3_^−^) dilution by the medium water produced acidosis of the same degree [7]. In the early 1980s Stewart [8] proposed an innovative model of acid–base balance regulation: changes in Cl^−^ concentration were suggested to be of particular importance, since it is the main negatively charged strong (fully dissociated) electrolyte in the extracellular space. According to Stewart’s approach pH is determined by three independent variables: (i) strong ion difference (SID), which is the difference between the sum of all strong cations (Na^+^, K^+^, Ca^2+^, Mg^2+^) and the sum of all strong anions (Cl^−^, other strong anions as lactate and ketones), (ii) partial pressure of carbon dioxide (PCO_2_) and (iii) concentrations of non-volatile weak acids (*A*_tot_), mainly albumin and phosphate. These variables change the degree of water dissociation into hydrogen and hydroxide ions according to three fundamental physicochemical principles that must be met simultaneously: (a) the law of mass preservation, (b) the law of electrical neutrality in aqueous solutions and thus in body fluids and (c) the law of mass action (i.e. the dissociation constant magnitude of weak electrolytes). The Cl^−^ concentration changes—due to either regulatory adaptations or underlying disorders of acid–base balance in the organism—may occur independently of sodium (Na^+^) changes. Na^+^ concentration is under hormonal control for the maintenance of plasma osmolality and water balance. Cl^−^ concentration can change by moving through cell membranes under the control of Donnan forces and by altered renal excretion depending on the acid–base status of the body [9]. Our knowledge about the function of Cl^−^ channels has grown considerably in recent years: for example renal excretion and reabsorption are mediated by Cl^−^ channels [10]; this knowledge has contributed to our understanding of the corresponding disorders by elucidating the pathophysiological mechanisms. In hyperchloraemia, increased Cl^−^ concentration decreases SID, which in turn leads to an increase in free H^+^ ions [8]. The importance of Stewart’s approach is clear in septic patients with low SID acidosis, who would have remained undiagnosed by changes in base excess, due to the alkalizing effect of hypoalbuminaemia [11].

However, there are indications that hyperchloraemia is not exclusively iatrogenic, but constitutes part of the pathophysiology of sepsis. Kellum and colleagues treated experimental shock (post-*E. coli* endotoxin infusion) in dogs with saline infusion. In this model it was demonstrated that the exogenous Cl^−^ administration could account for only one-third of the Cl^−^ serum increase, representing 38% of the total acid load; lactate contributed less than 10% of the acidic load at the end of the experimental period. The excess Cl^−^ was attributed to the activation of mechanisms that led to differential movements of Na^+^ and Cl^−^ from intracellular to extracellular spaces or from extravascular to intravascular compartments; the endothelial injury and the extravasation of albumin might represent a scenario for this Cl^−^ movement [12].

Hyperchloraemia has been studied in children with meningococcal septic shock and was found to be the main cause of metabolic acidosis post-resuscitation [13]; this observation was confirmed 2 years later in critically ill, adult patients with severe sepsis and septic shock [14]. Szrama and Smuszkiewicz [15], analysing 990 arterial blood gas results from 43 septic intensive care unit (ICU) patients, found that low SID acidosis (increased Cl^−^/Na^+^ ratio) was the most frequent cause of acidosis, being present in 93.5% of blood samples. In a pilot study from our centre, the most important cause of metabolic acidosis in septic patients was hyperchloraemia and low SID [16]. In particular, the causes of metabolic acidosis were investigated in 94 patients with sepsis and septic shock on the day of ICU admission. Cl^−^ levels were significantly elevated, resulting in a low SID, in the metabolic acidosis group (BE < − 2 mEq/L), while lactic acid levels did not differ significantly among the subgroups of patients with low (< − 2 mEq/L), normal (between − 2 and + 2 mEq/L) or elevated (> 2 mEq/L) base excess values. Of note is the fact that in this ICU balanced crystalloid solutions (Ringer’s lactate) are used for resuscitation, except for those cases requiring saline use (i.e. severe hyponatraemia).

### Hyperchloraemia and inflammatory response during sepsis

Sepsis represents a state of hyperimmune response to an infection, with both pro-inflammatory (systemic inflammatory response syndrome—SIRS) and anti-inflammatory (compensatory anti-inflammatory response syndrome—CARS) pathways being activated [17]. The possible effects of hyperchloraemic acidosis on the host’s immunity have been studied in experimental models.

Several studies [18–20] document that hydrochloric acid (HCl)-induced acidosis influences the TNF-α levels by enhancing TNF-α gene transcription [19]. However, a reduced TNF-α secretion is observed when pH reaches 7.0 or less [18] and the major pH-sensitive step(s) in TNF-α production appears to be located at a post-transcriptional level [21].

The upregulation of inducible nitric oxide synthase (iNOS) and the consequent overproduction of nitric oxide is associated with systemic hypotension and decreased vascular reactivity in septic patients [22]. Pedoto et al. [23, 24] reported the correlation of HCl-induced acidosis with increased iNOS activity, along with lung and intestinal injury in healthy rats. A previous study by Bellocq et al. [20] had suggested that lowering pH from 7.4 to 7.0 amplifies the nuclear factor-κB (NF-κB)-dependent iNOS pathway, by upregulating mRNA expression. However, extreme acidic conditions (pH 6.5) do not increase nitrite production [25], since the intracellular pH falls below 7.0, which has been suggested to be optimal for iNOS function [24].

The observed increase in both NF-κB binding activity in the nucleus and NF-κB-driven reporter gene expression under acidic conditions [20, 25] has been demonstrated to affect other inflammatory pathways. Kellum and co-workers studied the release of IL-6 and IL-10 in murine cell cultures stimulated with *E. coli* lipopolysaccharide after acidification with HCl. Extreme acidosis (pH 6.5) was associated with reduced release of both IL-6 and IL-10 as well as attenuated NF-κB DNA binding activity. However, the greater reduction in IL-10 levels led to a significant increase in IL-6 to IL-10 ratio from 5:1 at pH 7.4 to 55:1 at pH 6.5 [25]. In contrast to these in vitro results, data from experimental sepsis in rats suggest that both pro-inflammatory (TNF, IL-6) and anti-inflammatory (IL-10) mediators were increased by HCl infusion. The above results illustrate the major differences between ex vivo and in vivo models [26] due to a number of other parameters triggered by acidaemia (catecholamine synthesis, stimulation of vasopressin, adrenocorticotropic hormone and aldosterone).

In conclusion, hyperchloraemic acidification appears to have a main pro-inflammatory effect based on NO release, IL-6-to-IL-10 ratios and NF-κB binding activity demonstrated ex vivo. The reduced TNF-α secretion [18] and NF-κB binding activity [25] in severe acidosis suggest that not all pro-inflammatory pathways are preserved at the extremes of the acid–base homeostasis. In contrast, lactic acidosis appears to exhibit anti-inflammatory action by decreasing cytokine expression and NF-κB binding activity [25].

### Hyperchloraemia and haemodynamics

Severe acidosis in sepsis is associated with haemodynamic instability through many different pathophysiologic mechanisms (reduced left ventricular contractility, diastolic dysfunction and right ventricular failure, predisposition to cardiac arrhythmias, arterial vasodilation, impaired responsiveness to catecholamines, reduced hepatic blood flow, impaired oxygen tissue delivery) [27]. Kellum et al. [28] demonstrated that hyperchloraemic acidosis reduced the mean arterial pressure (MAP) in normotensive septic rats; the decrease in MAP showed a higher correlation to plasma Cl^−^ elevation as compared to pH reduction. Moderate acidosis enhanced plasma nitrite levels, a result confirming previous studies by Pedoto et al. [24] who reported an increase in serum nitrite levels and a significant decrease in blood pressure in healthy rats after HCl-induced acidosis.

### Hyperchloraemia and renal function

Among critically ill patients who develop acute kidney injury (AKI) almost 50% are septic. Furthermore, septic AKI is identified as an independent predictor of hospital death [29]. The administration of chloride-rich solutions and the probable adverse effects on kidney function have been studied in both animal and human trials.

An animal study performed by Wilcox indicated that intrarenal infusion of chloride-containing solutions led to renal vasoconstriction and fall in glomerular filtration rate (GFR). Chloride-induced vasoconstriction—which appeared to be specific for the renal vessels—was potentiated by previous salt depletion and related to tubular Cl^−^ reabsorption [30]. Pathophysiologically, this observation is attributed to high Cl^−^ sensitivity of K^+^-induced smooth muscle cell contraction in the afferent arterioles [31] and also to the activation of tubuloglomerular negative feedback provoked by higher levels of Cl^−^ transport across the macula densa cells [32]. The chloride-induced thromboxane release—and its actions on the afferent and efferent arterioles that contribute to renal vascular resistance—offers an additional, hypothetical mechanism for the fall in GFR during hyperchloraemia [33]. An experimental study by Quilley suggested that exposure of isolated rat kidney to higher Cl^−^ concentration increased vasoconstrictor responses to angiotensin II (Ang II) [34]. However, a previous study in greyhounds had shown that hyperchloraemia also decreases generation of Ang II, questioning the role of intrarenal renin–angiotensin system in chloride-induced renal vasoconstriction in intact animals [35].

Studies in animals [36] and human volunteers [37] have shown a correlation between higher levels of inflammatory markers in sepsis with the development of AKI. The observed increase in IL-6 levels following saline resuscitation in septic animals and its association with increased AKI risk provide an additional explanation for renal dysfunction during sepsis resuscitation treatment [38].

Moving from animal to human studies, Williams and colleagues conducted a trial in healthy human volunteers and reported that infusion of large volumes of 0.9% saline (50 mL/kg) within 1 h resulted in lower pH and significantly prolonged the time until first urination compared to infusion of Ringer’s lactate (RL) solution; the effect of acidosis persisted for 1 h beyond the end of infusion [39]. Other studies involving humans showed that the intravenous infusion of 2 L of 0.9% saline over 60 min resulted in a reduction in renal blood flow velocity and renal cortical tissue perfusion; such changes were not observed after infusion of balanced crystalloids [40]. The delayed time to first micturition in human volunteers, who had received 2 L intravenous infusions of 0.9% saline comparing to Hartman’s solution (within 1 h, on separate occasions), was associated with the development of hyperchloraemia in all subjects; hyperchloraemia was recorded for 6 h from the beginning of the infusion. In contrast, serum chloride concentrations remained normal after Hartmann’s infusion [41]. Although the infusion of a lower osmolality solution could relate to lower antidiuretic hormone secretion leading to earlier diuresis, the greater natriuresis observed could not support this theory [10]. A probable explanation might relate to chloride’s vasoconstrictive action, boosted by the previously reported slower excess chloride excretion during administration of saline solutions [42].

Most studies examining the pathophysiological effects of hyperchloraemia on kidney function have not been performed in septic patients exclusively, but include a heterogeneous population of ICU patients. In a meta-analysis of studies relating intravenous resuscitation fluids administration to patient outcomes in the perioperative or intensive care setting, high-chloride fluids were associated with a significantly higher risk of acute kidney injury [43]. Two out of the 21 meta-analysed trials, that included general ICU patients, gave conflicting results. Yunos and colleagues studied a rather diverse population (~ 50% post-operative, ~ 7% severe sepsis/septic shock patients) and demonstrated that chloride-restrictive intravenous fluids strategy was associated with a significant decrease in the incidence of (a) AKI, including the use of renal replacement therapy [44], (b) metabolic acidosis, (c) severe hyperchloraemia and (d) hypernatraemia [45]. On the contrary, the SPLIT trial (comparing saline vs balanced crystalloid) concluded that the type of crystalloid infused did not affect the incidence of AKI [46]. However, this study involved predominantly ICU post-operative population and sepsis was diagnosed in only 4% of the cases. In addition, the median volume of crystalloids infused was only 2000 cc, which might have affected the results as shown in the recently published SALT study. In this randomized trial on ICU patients the incidence of AKI did not differ between the two study arms (balanced crystalloids vs 0.9% saline). However, among patients who received larger volumes of fluids, those assigned to saline appeared to experience more major adverse kidney events [47]. The negative effect of the infused saline volume on kidney function has also been confirmed by two recent studies—a retrospective one by Sen et al. [48] and the SMART randomized trial [49]—involving critically ill patients. Furthermore, the SMART trial being a randomized study including 15,802 patients demonstrated a negative impact on renal function of saline infusion versus balanced crystalloids; this effect was even greater in the subgroup of 2336 septic patients.

Additional data from a retrospective cohort study suggest that maximal serum Cl^−^ concentration in the first 48 h after resuscitation of septic patients is associated with AKI. The increase in serum Cl^−^ exhibited a dose-dependent relationship with the severity of AKI, and remarkably, an increase in serum Cl^−^ ≥ 5 mmol/L was associated with the development of AKI even among patients who remained normochloraemic [50]. These results are consistent with those of an earlier retrospective analysis of data from general ICU population. Zhang et al. demonstrated—for the first time—that higher maximal and mean Cl^−^ concentration values were associated with the subsequent development of AKI, but the severity of AKI correlated only with the former [51]. Nevertheless, in a retrospective cohort study on septic patients published a year later [52] it was found that the prevalence of acute renal failure was independent of the crystalloid solution infused (balanced or isotonic saline). Therefore, large-scale randomized trials focusing on low versus high Cl^−^ strategy during fluid resuscitation in sepsis—as the ongoing FISSH trial [53]—must be undertaken for valid conclusions.

### Effects of hyperchloraemia on the gastrointestinal tract

Experimental models have demonstrated the association of HCl-induced metabolic acidosis with reduced gastric motility in pigs [54] and intestinal injury in rats [23]. A trial among elderly surgical patients revealed a significant trend towards increased nausea and vomiting when saline infusion was compared to balanced fluids; two-third of the saline-treated patient group developed hyperchloraemic metabolic acidosis [55]. This information and the abdominal discomfort provoked in healthy subjects by saline infusion [39] suggest that hyperchloraemic acidosis could be associated with complications such as gastroparesis, emesis and altered intestinal permeability; further investigation on this subject is required.

### Effects of hyperchloraemia on haemostasis and haemopoiesis

Coagulation activation during sepsis represents an important component of the overall response against invading pathogens [56]. This beneficial process can be reversed to a life-threatening event during severe inflammatory responses, since excessive thrombosis activation may lead to disseminated intravascular coagulation (DIC) and consumption of multiple clotting factors [57]. The correlation of hyperchloraemia during sepsis with coagulation has not been studied yet. A limited number of experimental and clinical studies [58, 59] suggest that large volumes of chloride-rich solutions lead to coagulation disorders and increased tendency for bleeding. However, coagulation abnormalities in substantial blood loss and haemorrhagic shock (and this was the case in the above-mentioned studies) are influenced by several factors—hormonal, immunological, extent of blood loss, hypoxia, acidosis, hypothermia—[60] that differ or may not be present in septic patients. On the other hand, the reduced coagulation proteases’ activity observed in acidic pH [61] could affect the progression of sepsis in patients with hyperchloraemic acidosis by undermining coagulation activation; this hypothesis should be further investigated.

Anaemia is common in septic patients and is caused by shortening of red blood cell (RBC) circulatory lifespan (haemolysis, phlebotomy losses, invasive procedures, gastrointestinal bleeding, etc.) and/or diminished RBC production due to nutritional deficiencies, iron metabolism, inflammatory processes leading to impaired RBC proliferation, impaired erythropoietin production and signalling [62]. The results of a cohort study by Neyra et al. [63] in septic patients suggest that anaemia is more frequent in hyperchloraemic patients, requiring more blood transfusions compared to normochloraemic patients. A probable mechanism explaining this observation might relate to inhibition of erythropoietin production by pro-inflammatory cytokines [64] since the latter predominate in hyperchloraemic acidosis [25].

### Hyperchloraemia and mortality in sepsis

The negative impact of hyperchloraemia on the mortality of septic [14] and critically ill [65] patients was first reported less than a decade ago. In contrast, data from a single-centre observational retrospective cohort study of critically ill septic patients suggested that Cl^−^ levels upon ICU admission were not related to hospital mortality. However, in subjects with deteriorating hyperchloraemia, higher levels of Cl^−^ (≥ 5 mEq/L) 72 h later were associated with increased hospital mortality [63]. Since the study population was limited to ICU patients, the acid–base balance could have been manipulated by fluid administration in the emergency room. Rochwerg et al. performed a meta-analysis of the effect different resuscitative fluids (crystalloids and colloids) had on the mortality rate in septic patients. A trend towards improved survival of patients resuscitated with balanced crystalloids compared to patients who received saline was noted at a 6-node meta-analysis level (crystalloids vs albumin vs hydroxyethyl starch vs gelatin, with crystalloids divided into balanced or unbalanced and hydroxyethyl starch divided into low or high molecular weight) [66]. Studies that compared chloride-rich versus balanced crystalloid solutions were designed and confirmed the previous findings. Shaw and colleagues conducted a retrospective cohort study that demonstrated an association between lower Cl^−^ load and lower mortality in hospitalized patients with SIRS; this observation was independent of the total fluid volume administered and remained significant after adjustment for illness severity [67]. Raghunathan et al. [52] confirmed the previous observation in septic patients who received greater proportions of balanced crystalloids. The retrospective study of Sen et al. [48] showed a negative association between higher Cl^−^ load and survival rate among ICU patients who had received large-volume fluids; this effect extended to at least 1 year post-ICU admission. The SMART randomized trial demonstrated a statistically significant higher 30-day in-hospital mortality in septic patients treated with saline compared to those treated with balanced crystalloids [49].

### Chloride-restrictive fluid administration

Saline 0.9% is the most commonly used crystalloid solution globally [50], even though doubts regarding its physiological effects had been raised as early as the end of the nineteenth century [68]; these observations led to the production of the first buffered solutions. The drastic changes in the pathophysiology of metabolic acidosis in sepsis within 8 h post-aggressive isotonic fluid resuscitation—from acidosis due to unmeasured anions to hyperchloraemic acidosis—[13] is an effect many clinicians are not aware of. This common condition among ICU patients [69] has probably influenced the clinical practice of intensivists, who favour the use of buffered crystalloid solutions over saline according to the FENICE study [70]. The latest Surviving Sepsis Campaign guidelines do not recommend balanced crystalloids over saline; however, close monitoring of chloride levels is advised for avoidance of hyperchloraemia [71]. Raised concerns about iatrogenic metabolic acidosis in critically ill patients are fully justified, because of the inadequate respiratory compensation and the anticipated exhaustion of buffering reserves [12]. It should be noted that from the studies mentioned, it cannot be safely ascertained whether the adverse events observed in the case of hyperchloraemia are due to elevated levels of Cl^−^ or acidosis per se.

The beneficial effect of balanced crystalloids is supported by the probable harmful effects of saline on MAP, renal haemodynamics and the gastrointestinal tract, particularly in the setting of sepsis-induced systemic hypoperfusion and lastly the higher mortality rates of patients receiving saline [49, 72]. Furthermore, among the ten studies (involving 15,000 critically ill patients) subjected to meta-analysis, none demonstrated superiority of saline in organ function or mortality rate over balanced crystalloids [43, 73]. The lower transfusion rate in patients receiving low-chloride balanced fluids supports the chloride-restrictive practice, although an incompatibility of calcium-containing buffered solutions and citrated blood cannot be excluded [43]. Buffered solutions have been associated with lower prevalence of SIRS and C-reactive protein levels—compared to saline in patients with acute pancreatitis [74]—and a lower risk of post-operative infections after open abdominal surgeries [75]; such observations are compatible with the pro-inflammatory effects of saline-induced hyperchloraemic acidosis demonstrated in experimental models. Up to date, saline remains the fluid of choice in septic patients with comorbidities such as severe hyponatraemia, cerebral oedema and brain injury [76].

## Conclusions

Chloride, as the major anion of the extracellular fluid, constitutes an important element in the homeostasis of the human organism. Hyperchloraemia, whether a result of the sepsis process or a consequence of its treatment with supraphysiologic chloride fluids, appears to have a negative impact on the clinical outcome of septic patients. The detrimental effect of hyperchloraemic acidosis on the inflammatory response, on haemodynamics and also upon the homeostasis of organs or systems, demonstrated by studies in humans and some experimental models of sepsis, should keep clinicians alert. Close monitoring of chloride levels and acid–base homeostasis pertains to all levels of hospitalization, starting with the early resuscitation treatment in the emergency room. Balanced crystalloids appear to improve the sepsis outcome, when compared to saline. Large-scale randomized trials analysing more than one endpoint (mortality, haemodynamics, AKI, haemostatic disorders, other organ damage, length of ICU and hospital stay) are urgently needed in order to confirm the possible beneficial effect of chloride restriction strategies.

## Abbreviations

Cl^−^: chloride
SID: strong ion difference
PCO_2_: partial pressure of carbon dioxide
*A*_tot_: non-volatile weak acids
Na^+^: sodium
ICU: intensive care unit
SIRS: systemic inflammatory response syndrome
CARS: compensatory anti-inflammatory response syndrome
HCl: hydrochloric acid
iNOS: inducible nitric oxide synthase
NF-κB: nuclear factor-κB
MAP: mean arterial pressure
AKI: acute kidney injury
GFR: glomerular filtration rate
Ang II: angiotensin II
RL: Ringer’s lactate
DIC: disseminated intravascular coagulation
RBC: red blood cell

## References

[1] Liu V, Escobar GJ, Greene JD, Soule J, Whippy A, Angus DC (2014). Hospital deaths in patients with sepsis from 2 independent cohorts. JAMA.

[2] Berend K, Van Hulsteijn LH, Gans ROB (2012). Chloride: the queen of electrolytes?. Eur J Intern Med.

[3] Tani M, Morimatsu H, Takatsu F, Morita K (2012). The incidence and prognostic value of hypochloremia in critically ill patients. Sci World J.

[4] Kellum JA (2004). Metabolic acidosis in patients with sepsis: epiphenomenon or part of the pathophysiology?. Crit Care Resusc.

[5] Van Slyke DD, Wu H, McLean FC (1923). Studies of gas and electrolyte equilibria in the blood. V. Factors controlling the electrolyte and water distribution in the blood. J Biol Chem.

[6] Shires GT, Holman J (1948). Dilution acidosis. Ann Intern Med.

[7] Asano S, Kato E, Yamauchi M, Ozawa Y, Iwasa M, Wada T (1966). The mechanism of the acidosis caused by infusion of saline solution. Lancet.

[8] Stewart PA (1983). Modern quantitative acid–base chemistry. Can J Physiol Pharmacol.

[9] Stewart PA, Kellum JA, Elbers P (2009). Whole-body acid–base balance. Stewart’s textbook of acid–base.

[10] Yunos NM, Bellomo R, Story D, Kellum J (2010). Bench-to-bedside review: chloride in critical illness. Crit Care.

[11] Mallat J, Michel D, Salaun P, Thevenin D, Tronchon L (2012). Defining metabolic acidosis in patients with septic shock using Stewart approach. Am J Emerg Med.

[12] Kellum JA, Bellomo R, Kramer DJ, Pinsky MR (1998). Etiology of metabolic acidosis during saline resuscitation in endotoxemia. Shock.

[13] O’Dell E, Tibby SM, Durward A, Murdoch IA (2007). Hyperchloremia is the dominant cause of metabolic acidosis in the postresuscitation phase of pediatric meningococcal sepsis. Crit Care Med.

[14] Noritomi DT, Soriano FG, Kellum JA, Cappi SB, Biselli PJC, Libório AB (2009). Metabolic acidosis in patients with severe sepsis and septic shock: a longitudinal quantitative study. Crit Care Med.

[15] Szrama J, Smuszkiewicz P (2016). An acid–base disorders analysis with the use of the Stewart approach in patients with sepsis treated in an intensive care unit. Anesthesiol Intensive Ther.

[16] Vasileiadis I, Kompoti M, Tripodaki ES, Rovina N, Pontikis K, Ntouka E (2017). Metabolic acidosis in patients with sepsis. ESICM LIVES 2017. Intensive Care Med Exp.

[17] Ward NS, Casserly B, Ayala A (2008). The compensatory anti-inflammatory response syndrome (CARS) in critically ill patients. Clin Chest Med.

[18] Bidani A, Wang C-Z, Saggi SJ, Heming TA (1998). Evidence for pH sensitivity of tumor necrosis factor-alpha release by alveolar macrophages. Lung.

[19] Heming TA, Davé SK, Tuazon DM, Chopra AK, Peterson JW, Bidani A (2001). Effects of extracellular pH on tumour necrosis factor-alpha production by resident alveolar macrophages. Clin Sci.

[20] Bellocq A, Suberville S, Philippe C, Bertrand F, Perez J, Fouqueray B (1998). Low environmental pH is responsible for the induction of nitric-oxide synthase in macrophages. Evidence for involvement of nuclear factor-kappaB activation. J Biol Chem.

[21] Heming TA, Tuazon DM, Davé SK, Chopra AK, Peterson JW, Bidani A (2001). Post-transcriptional effects of extracellular pH on tumour necrosis factor-a production in RAW 246.7 and J774 A.1 cells. Clin Sci.

[22] Nava E, Palmer RMJ, Moncada S (1991). Inhibition of nitric oxide synthesis in septic shock: how much is beneficial?. Lancet.

[23] Pedoto A, Nandi J, Oler A, Camporesi EM, Hakim TS, Levine RA (2001). Role of nitric oxide in acidosis-induced intestinal injury in anesthetized rats. J Lab Clin Med.

[24] Pedoto A, Caruso JE, Nandi J, Oler A, Hoffman SP, Tassioupolos AK (1999). Acidosis stimulates nitric oxide production and lung damage in rats. Am J Respir Crit Care Med.

[25] Kellum JA, Song M, Li J (2004). Lactic and hydrochloric acids induce different patterns of inflammatory response in LPS-stimulated RAW 264.7 cells. AJP Regul Integr Comp Physiol.

[26] Kellum JA, Song M, Almasri E (2006). Hyperchloremic acidosis increases circulating inflammatory molecules in experimental sepsis. Chest.

[27] Velissaris D, Karamouzos V, Ktenopoulos N, Pierrakos C, Karanikolas M (2015). The use of sodium bicarbonate in the treatment of acidosis in sepsis: a literature update on a long term debate. Crit Care Res Pract.

[28] Kellum JA, Song M, Venkataraman R (2004). Effects of hyperchloremic acidosis on arterial pressure and circulating inflammatory molecules in experimental sepsis. Chest.

[29] Bagshaw SM, Uchino S, Bellomo R, Morimatsu H, Morgera S, Schetz M (2007). Septic acute kidney injury in critically ill patients: clinical characteristics and outcomes. Clin J Am Soc Nephrol.

[30] Wilcox CS (1983). Regulation of renal blood flow by plasma chloride. J Clin Invest.

[31] Hansen PB, Jensen BL, Skott O (1998). Chloride regulates afferent arteriolar contraction in response to depolarization. Hypertension.

[32] Schnermann J, Ploth DW, Hermle M (1976). Activation of tubulo-glomerular feedback by chloride transport. Pflugers Arch.

[33] Bullivant EM, Wilcox CS, Welch WJ, Mary E, Bullivant A, Wilcox CS (1989). Intrarenal vasoconstriction during hyperchloremia: role of thromboxane. Am J Physiol Renal Physiol.

[34] Quilley CP, Lin YR, McGiff JC (1993). Chloride anion concentration as a determinant of renal vascular responsiveness to vasoconstrictor agents. Br J Pharmacol.

[35] Wilcox CS, Peart WS (1987). Release of renin and angiotensin II into plasma and lymph during hyperchloremia. Am J Physiol.

[36] Peng Z-Y, Wang H-Z, Srisawat N, Wen X, Rimmelé T, Bishop J (2012). Bactericidal antibiotics temporarily increase inflammation and worsen acute kidney injury in experimental sepsis. Crit Care Med.

[37] Murugan R, Karajala-Subramanyam V, Lee M, Yende S, Kong L, Carter M (2010). Acute kidney injury in non-severe pneumonia is associated with an increased immune response and lower survival. Kidney Int.

[38] Zhou F, Peng Z-Y, Bishop JV, Cove ME, Singbartl K, Kellum JA (2014). Effects of fluid resuscitation with 0.9% saline versus a balanced electrolyte solution on acute kidney injury in a rat model of sepsis. Crit Care Med.

[39] Williams EL, Hildebrand KL, McCormick SA, Bedel MJ (1999). The effect of intravenous lactated Ringer’s solution versus 0.9% sodium chloride solution on serum osmolality in human volunteers. Anesth Analg.

[40] Chowdhury AH, Cox EF, Francis ST, Lobo DN (2012). A, randomized, controlled, double-blind crossover study on the effects of 2-L infusions of 0.9% saline and plasma-lyte^®^ 148 on renal blood flow velocity and renal cortical tissue perfusion in healthy volunteers. Ann Surg.

[41] Reid F, Lobo DN, Williams RN, Rowlands BJ, Allison SP (2003). (Ab)normal saline and physiological Hartmann’s solution: a randomized double-blind crossover study. Clin Sci.

[42] Veech RL (1986). The toxic impact of parenteral solutions on the metabolism of cells: a hypothesis for physiological parenteral therapy. Am J Clin Nutr.

[43] Krajewski ML, Raghunathan K, Paluszkiewicz SM, Schermer CR, Shaw AD (2015). Meta-analysis of high- versus low-chloride content in perioperative and critical care fluid resuscitation. Br J Surg.

[44] Yunos NM, Bellomo R, Hegarty C, Story D, Ho L, Bailey M (2012). Association between a chloride-liberal vs chloride-restrictive intravenous fluid administration strategy and kidney injury in critically ill adults. JAMA.

[45] Yunos NM, Kim IB, Bellomo R, Bailey M, Ho L, Story D (2011). The biochemical effects of restricting chloride-rich fluids in intensive care. Crit Care Med.

[46] Young P, Bailey M, Beasley R, Henderson S, Mackle D, McArthur C (2015). Effect of a buffered crystalloid solution vs saline on acute kidney injury among patients in the intensive care unit: the SPLIT Randomized Clinical Trial. JAMA.

[47] Semler MW, Wanderer JP, Ehrenfeld JM, Stollings JL, Self WH, Siew ED (2017). Balanced crystalloids versus saline in the intensive care unit. The SALT Randomized Trial. Am J Respir Crit Care Med.

[48] Sen A, Keener CM, Sileanu FE, Foldes E, Clermont G, Murugan R (2017). Chloride content of fluids used for large-volume resuscitation is associated with reduced survival. Crit Care Med.

[49] Semler MW, Self WH, Wanderer JP, Ehrenfeld JM, Wang L, Byrne DW (2018). Balanced crystalloids versus saline in critically ill adults. N Engl J Med.

[50] Suetrong B, Pisitsak C, Boyd JH, Russell JA, Walley KR (2016). Hyperchloremia and moderate increase in serum chloride are associated with acute kidney injury in severe sepsis and septic shock patients. Crit Care.

[51] Zhang Z, Xu X, Fan H, Li D, Deng H (2013). Higher serum chloride concentrations are associated with acute kidney injury in unselected critically ill patients. BMC Nephrol.

[52] Raghunathan K, Shaw A, Nathanson B, Stürmer T, Brookhart A, Stefan MS (2014). Association between the choice of IV crystalloid and in-hospital mortality among critically ill adults with sepsis. Crit Care Med.

[53] Rochwerg B, Millen T, Austin P, Zeller M, D’Aragon F, Jaeschke R (2017). Fluids in Sepsis and Septic Shock (FISSH): protocol for a pilot randomised controlled trial. BMJ Open.

[54] Tournadre JP, Allaouchiche B, Malbert CH, Chassard D (2000). Metabolic acidosis and respiratory acidosis impair gastro-pyloric motility in anesthetized pigs. Anesth Analg.

[55] Wilkes NJ, Woolf R, Mutch M, Mallett SV, Peachey T, Stephens R (2001). The effects of balanced versus saline-based hetastarch and crystalloid solutions on acid–base and electrolyte status and gastric mucosal perfusion in elderly surgical patients. Anesth Analg.

[56] Fiusa MML, Carvalho-Filho MA, Annichino-Bizzacchi JM, De Paula EV (2015). Causes and consequences of coagulation activation in sepsis: an evolutionary medicine perspective. BMC Med.

[57] Levi M, van der Poll T (2010). Inflammation and coagulation. Crit Care Med.

[58] Todd SR, Malinoski D, Muller PJ, Schreiber MA (2007). Lactated Ringer’s is superior to normal saline in the resuscitation of uncontrolled hemorrhagic shock. J Trauma Inj Infect Crit Care.

[59] Waters JH, Gottlieb A, Schoenwald P, Popovich MJ, Sprung J, Nelson DR (2001). Normal saline versus lactated Ringer’s solution for intraoperative fluid management in patients undergoing abdominal aortic aneurysm repair: an outcome study. Anesth Analg.

[60] Cap A, Hunt BJ (2015). The pathogenesis of traumatic coagulopathy. Anaesthesia.

[61] Meng ZH, Wolberg AS, Monroe DM, Hoffman M (2003). The effect of temperature and ph on the activity of factor VIIa: implications for the efficacy of high-dose factor VIIa in hypothermic and acidotic patients. J Trauma Inj Infect Crit Care.

[62] Hayden SJ, Albert TJ, Watkins TR, Swenson ER (2012). Anemia in critical illness: insights into etiology, consequences, and management. Am J Respir Crit Care Med.

[63] Neyra JA, Canepa-Escaro F, Li X, Manllo J, Adams-Huet B, Yee J (2015). Association of hyperchloremia with hospital mortality in critically ill septic patients. Crit Care Med.

[64] Jelkmann W (1998). Proinflammatory cytokines lowering erythropoietin production. J Interf Cytokine Res..

[65] Boniatti MM, Cardoso PRC, Castilho RK, Vieira SRR (2011). Is hyperchloremia associated with mortality in critically ill patients? A prospective cohort study. J Crit Care.

[66] Rochwerg B, Alhazzani W, Sindi A, Heels-Ansdell D, Thabane L, Fox-Robichaud A (2014). Fluid resuscitation in sepsis: a systematic review and network meta-analysis. Ann Intern Med.

[67] Shaw AD, Raghunathan K, Peyerl FW, Munson SH, Paluszkiewicz SM, Schermer CR (2014). Association between intravenous chloride load during resuscitation and in-hospital mortality among patients with SIRS. Intensive Care Med.

[68] Hahn RG (2014). Should anaesthetists stop infusing isotonic saline?. Br J Anaesth.

[69] Klemz K, Ho L, Bellomo R (2008). Daily intravenous chloride load and the acid–base and biochemical status of intensive care unit patients. J Pharm Pract Res..

[70] Cecconi M, Hofer C, Teboul J-L, Pettila V, Wilkman E, Molnar Z (2015). Fluid challenges in intensive care: the FENICE study. Intensive Care Med.

[71] Rhodes A, Evans LE, Alhazzani W, Levy MM, Antonelli M, Ferrer R (2017). Surviving sepsis campaign. Crit Care Med.

[72] Rochwerg B, Alhazzani W, Gibson A, Ribic CM, Sindi A, Heels-Ansdell D (2015). Fluid type and the use of renal replacement therapy in sepsis: a systematic review and network meta-analysis. Intensive Care Med.

[73] Semler MW, Rice TW (2016). Saline is not the first choice for crystalloid resuscitation fluids. Crit Care Med.

[74] Wu BU, Hwang JQ, Gardner TH, Repas K, Delee R, Yu S (2011). Lactated Ringer’s solution reduces systemic inflammation compared with saline in patients with acute pancreatitis. Clin Gastroenterol Hepatol.

[75] Shaw AD, Bagshaw SM, Goldstein SL, Scherer LA, Duan M, Schermer CR (2012). Major complications, mortality, and resource utilization after open abdominal surgery. Ann Surg.

[76] Myburgh JA, Mythen MG (2013). Resuscitation fluids. N Engl J Med.

